# Measurement of Fumonisins in Maize Using a Portable Mass Spectrometer

**DOI:** 10.3390/toxins14080523

**Published:** 2022-07-30

**Authors:** Chris M. Maragos, Kristin Barnett, Luke Morgan, Martha M. Vaughan, Kristal K. Sieve

**Affiliations:** 1 Mycotoxin Prevention and Applied Microbiology Research Unit, National Center for Agricultural Utilization Research, Agricultural Research Service, USDA, 1815 N University, Peoria, IL 61604, USA; martha.vaughan@usda.gov (M.M.V.); kristal.sieve@usda.gov (K.K.S.); 2 Agricultural Products Inspection, Illinois Department of Agriculture, 801 E. Sangamon Avenue, Springfield, IL 62702, USA; kristin.barnett@illinois.gov (K.B.); luke.morgan@illinois.gov (L.M.)

**Keywords:** mycotoxin, fumonisin, analysis, portable mass spectrometry, maize

## Abstract

Fumonisins are a group of mycotoxins that routinely contaminate maize. Their presence is monitored at multiple stages from harvest to final product. Immunoassays are routinely used to screen commodities in the field while laboratory-based methods, such as mass spectrometry (MS), are used for confirmation. The use of a portable mass spectrometer unlocks the potential to conduct confirmatory analyses outside of traditional laboratories. Herein, a portable mass spectrometer was used to measure fumonisins in maize. Samples were extracted with aqueous methanol, cleaned up on an immunoaffinity column, and tested with the portable MS. The limits of detection were 0.15, 0.19, and 0.28 mg/kg maize for fumonisins B_1_ (FB_1_), FB_2_/FB_3_, and total fumonisins, respectively. The corresponding limits of quantitation in maize were 0.33, 0.59, and 0.74 mg/kg. Recoveries ranged from 93.6% to 108.6%. However, RSDs ranged from 12.0 to 29.8%. The method was applied to the detection of fumonisins in 64 samples of maize collected as part of the Illinois Department of Agriculture’s monitoring program. Good correlations were observed between the portable MS and a laboratory-based LC-MS method (r^2^ from 0.9132 to 0.9481). Results suggest the portable MS can be applied to the measurement of fumonisins in maize at levels relevant to international regulations.

## 1. Introduction

**Figure 1:**
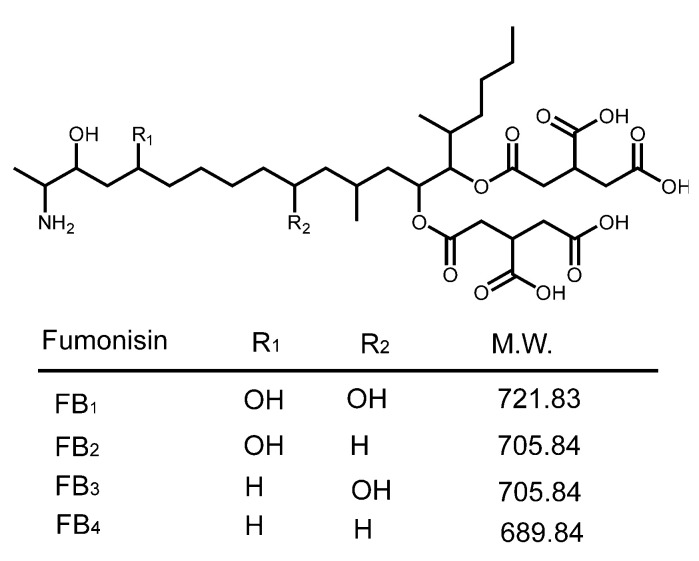
Structures of certain of the fumonisins with their R-group substituents.

Fungal infections are responsible for a variety of diseases in crops used for human food or animal feed. Disease of the plant is one outcome of fungal infection. However, certain fungi may also produce secondary metabolites, mycotoxins, that are toxic to animals, including humans. One group of mycotoxins, the fumonisins, have been implicated as causative agents of leukoencephalomalacia in horses, pulmonary edema in swine [1,2,3], and have been associated with neural tube defects in humans [4]. Fumonisin B_1_ (FB_1_) was classified by the International Agency for Research on Cancer as Group 2B; possibly carcinogenic to humans [5]. As a group, the fumonisins are characterized by a long carbon ‘backbone’ substituted with one or more hydroxyl groups and one or more tricarballylic acid groups. The various congeners are distinguished by the number and location of the substituents. The most prevalent member of the family is fumonisin B_1_ (FB_1_), which constitutes about 70% of the fumonisins found in naturally contaminated maize [1]. Maize generally contains smaller, but significant amounts of fumonisin B_2_ (FB_2_), and lesser amounts of fumonisins B_3_ and B_4_ (FB_3_, FB_4_) (Figure 1). The fumonisins are produced mainly by *Fusarium verticillioides* and *Fusarium proliferatum*, which infest maize worldwide [6]. The fungi may exist endophytically within the growing maize plant, but often cause diseases such as stalk rot or ear rot. In animals, the toxicity of the fumonisins has been attributed to the disruption of sphigolipid metabolism [7,8]. The early history of the fumonisins and their relevance to human and animal health were reviewed by Marasas [2]. A more recent review of the toxicity and mechanisms of action has been provided by Voss et al. [8].

The potential effects of fumonisins on human and animal health have resulted in the promulgation of regulatory or guidance levels for these toxins in many countries around the world. In the United States, the Food and Drug Administration (USFDA) has established guidance levels for total fumonisins in maize products, in foods, and in feeds. The suggested maximum levels vary depending upon the animal species and the proportion of contaminated material in the total diet. Guidance levels range between 2 and 4 mg/kg for human foods and between 5 to 100 mg/kg for animal feeds [9].

The fumonisins were identified many years ago. Since then, a great variety of analytical methods have been developed for their detection. Rapid screening of samples is often conducted at grain storage facilities using immunoassays. These include a variety of commercially available enzyme linked immunosorbent assays (ELISAs) and immunochromatographic devices such as ‘dipsticks’, lateral flow immunoassays, and biosensors [10,11,12,13,14,15]. The fumonisins lack a chromophore, and for this reason, instrumental methods, such as liquid chromatography (LC) that rely upon derivatization with a fluorophore have been traditionally used [16,17,18,19,20,21]. Since the 1990’s a great number of LC-mass spectrometry (LC-MS) and LC-MS/MS methods have been developed for quantitation of fumonisins in commodities, food products, and biological samples [22,23,24,25,26,27,28,29]. Most such methods use electrospray injection (ESI) with the instrument operated in positive mode. Single ion monitoring typically measures [M + H]^+^ [22,24,29]. The cleanup used has varied from simple centrifugation and filtration of extracts to the use of immunoaffinity cleanup columns (IAC) [22,23,24,25,26,27,28,29]. More recent contributions to the literature in this area were reviewed by De Girolamo et al. [30] and the topic is covered annually by Tittlemier et al. [31]. Many of the more recent contributions are multi-mycotoxin MS/MS methods that include the use of stable isotope internal standards to improve quantitation [32,33]. Advanced LC-MS methods for mycotoxins, including fumonisins, were reviewed by Malachová et al. [34]. Some interesting innovations in fumonisin detection involve MALDI imaging of these toxins in moldy foods [35], application of capillary electrophoresis-MS [36], and molecular imprinted polymer paper spray MS [37].

Improvements to mass spectrometers have progressed in many directions, including portability. Portable instruments are of great interest for monitoring hazardous materials in situ, such as chemical warfare agents, explosives, illicit drugs, alkaloids in plant tissues, agricultural chemicals, and forensic samples [38,39]. Some background on early efforts to miniaturize MS instruments was provided by Ouyang et al. [40]. Since that time, commercial fit-for-purpose instruments have become available that are more rugged and portable. Examples include GC-MS instruments such as the Torion-9 (Perkin-Elmer, Waltham, MA, USA) and the Griffin G460 (Teldyne FLIR LLC, Wilsonville, OR, USA). Portable instruments that use inlets other than gas chromatography include the MT Explorer 50 (Mass Tec Inc., Columbia, MD, USA) and the Portability (BaySpec, Inc., San Jose, CA, USA). Testing for mycotoxins occurs throughout the supply chain, and the ability to monitor for their presence with devices that can be used outside of the traditional analytical laboratory is necessary. Much of this type of monitoring is currently conducted with indirect methods such as immunoassays. The objective of this research was to determine the potential for direct methods, such as portable MS to be used in such situations, in particular for the determination of fumonisins in maize at levels sufficient to meet U.S. guidelines.

## 2. Results

### 2.1. Sample Preparation and Collection of Spectra

**Figure 2:**
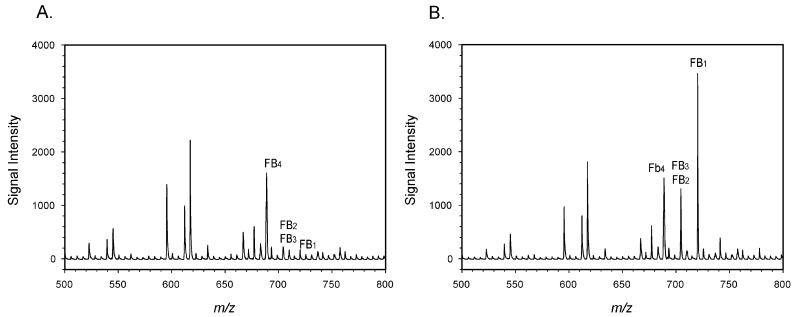
(**A**) unspiked maize; (**B**) maize spiked with 2.8 mg/kg total fumonisins (1.91 mg/kg FB_1_, 0.36 mg/kg FB_2_, and 0.56 mg/kg FB_3_). FB_4_ was added to both as an internal standard.

Maize samples were extracted with a mixture of aqueous methanol, the extracts were diluted with buffer and the fumonisins were isolated with a commercial immunoaffinity column (IAC). Eluate from the IAC was spiked with FB_4_, which was used as an internal standard. This mixture was infused into the instrument, with spectra collected over the range of 50–850 *m/z*. The signal from FB_1_ was continuously monitored and the spectrum collected 100 sec from the start was used for measurement. Spectra from a ‘blank’ (unspiked) sample and a sample spiked with the equivalent of 2.8 mg/kg total fumonisins are shown in Figure 2.

Because FB_2_ and FB_3_ have the same molecular weight, the signal at *m/z* = 705 was the sum of the two toxins. The spectra of the blank displayed small signals at the *m/z* corresponding to FB_1_ and FB_2_/FB_3_, as evidenced by the background noise in Figure 2A. While it can be difficult to obtain maize truly ‘free’ of FB_1_, the unspiked maize had been previously tested and found to contain less than 0.1 mg/kg FB_1_ by LC-MS. Small peaks at *m/z* 705 and 721, equivalent to those seen in Figure 2A were also observed in solvent blanks (not shown). For this reason, the signals in Figure 2A at these *m/z* were attributed to background and not to FB_1_, FB_2_, or FB_3_ in the sample.

### 2.2. Performance Criteria

**Figure 3:**
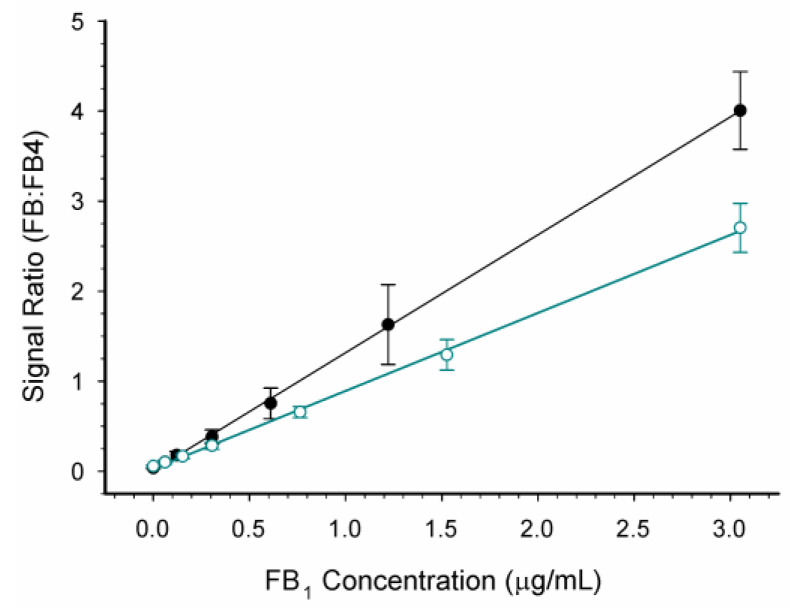
Effect of maize matrix on fumonisin detection with the portable MS. Calibration curves were prepared either in solvent (closed circles) or purified maize matrix (open circles). Data shown are the averages with ± 1 standard deviation (*n* = 5 for solvent, *n* = 20 for matrix).

Spectra were collected from mixtures of FB_1_, FB_2_/FB_3_, and FB_4_ at a variety of concentrations in both a solvent mixture (acetonitrile/H_2_O/formic acid) and in a purified matrix obtained from unspiked maize (i.e., matrix-matched). The data were fit with first order regression (Figure 3).

Calibration curves collected on either 5 separate days (in solvent) or 20 separate days (in a matrix) are depicted in Figure 3. The regression line of data collected in solvent was Y = 0.0083 + 1.3087(X), with r^2^ = 0.9996. The regression line of data collected in matrix-matched standards was Y = 0.0274 + 0.8656(X), with r^2^ = 0.9988. Regression fits for FB_2_/FB_3_ and total FB in a matrix are provided in Supplemental Figure S1. The magnitude of a matrix effect is often calculated by dividing the slope of the calibration curve in a matrix by that of the calibration curve in a solvent. The ratio, 66%, indicates ionization of FB_1_ was suppressed in the presence of purified matrix.

**Figure 4:**
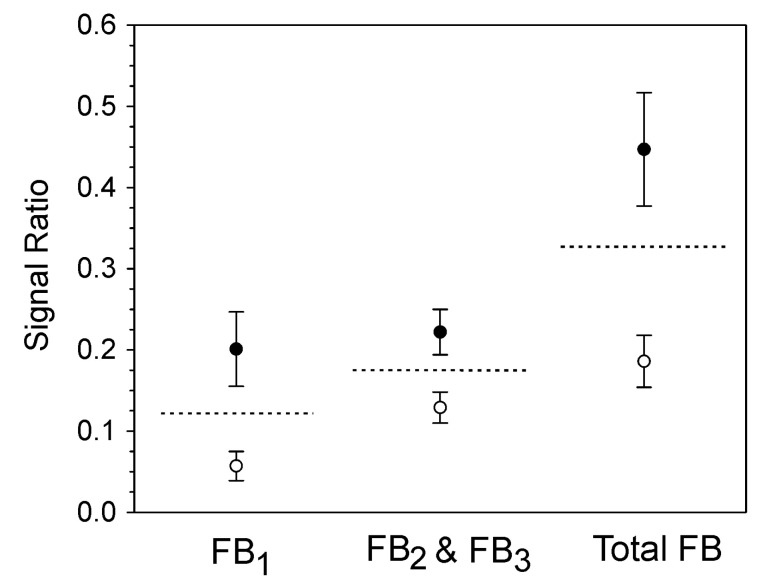
Discrimination between spiked (filled circles) and unspiked (open circles) maize samples. Spiked samples contained 0.57 µg/g added fumonisins (0.38 mg/kg FB_1_, 0.08 mg/kg FB_2_ and 0.11 mg/kg FB_3_). FB_2_ and FB_3_, which have the same *m/z* were recorded together. Values are presented as the average of 24 replicates collected over 6 days. Error bars are ± 1 standard deviation. Dashed lines represent the calculated Cut-Off ratios, representing 95% confidence for false negative determination.

The European Union (EU) has established criteria for the determination of ‘cut-off values’ for screening assays (Commission Regulation EU No.519/2014) [41]. Determining the cut-off level requires selecting a target concentration (STC) at which the assay will be used. In this work, the STC was selected as 0.57 mg/kg total fumonisins, equal to a sample containing 0.38 mg/kg FB_1_ and 0.19 mg/kg of combined FB_2_/FB_3_. The EU criteria use the average signals and error estimates from 20 or more spiked samples, collected on 5 or more separate days, to establish the concentration at which the false negative rate will be 5% at the STC. Graphic representations of the averages and error estimates used in the calculation are depicted in Figure 4. The cut-off ratios, calculated as signal ratios (i.e., relative to the internal standard) were 0.122, 0.174, and 0.327 for FB_1_, FB_2_/FB_3_, and total FB respectively. From the calibration curves, these ratios correspond to toxin concentrations of 0.22 mg/kg, 0.13 mg/kg, and 0.42 mg/kg for FB_1_, FB_2_/FB_3_, and total FB, respectively. At these concentrations, the false negative rate was predicted to be 5%.

Forty-five samples of maize were spiked with fumonisins, at levels ranging from 0.38 to 3.8 mg/kg, 0.19 to 1.85 mg/kg, and 0.57 to 5.66 mg/kg for FB_1_, FB_2_/FB_3_, and total FB respectively. Recoveries were high, ranging from 93.6% to 108.6% (Table 1). Unfortunately, variability was also high, with RSDs ranging from 12.0 to 29.8%.

Other performance criteria, such as limit of detection (LOD), and limit of quantification (LOQ) were also determined. The LOD was determined from error estimates associated with unspiked samples: 3 SD above the blank values for LOD and 10 SD above the blank values for LOQ. The LODs in maize were 0.15, 0.19, and 0.28 mg/kg for FB_1_, FB_2_/FB_3_, and total FB, respectively. The corresponding LOQs in maize were 0.33, 0.59, and 0.74 mg/kg.

### 2.3. Naturally Contaminated Samples

**Figure 5:**
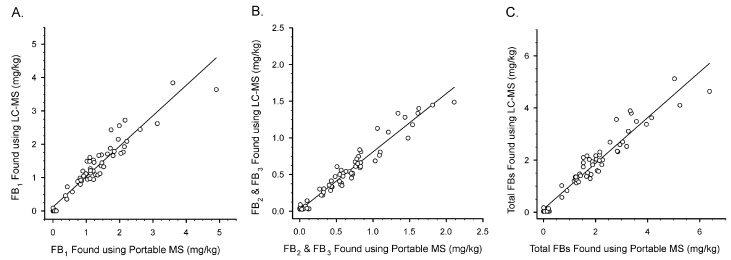
Comparison between the portable MS and LC-MS for 64 samples of Illinois maize. (**A**) FB_1_ alone; (**B**) FB_2_ and FB_3_ combined; (**C**) ‘Total’ fumonisins (FB_1_ + FB_2_ + FB_3_). Lines shown are first order regression fits.

The Illinois Department of Agriculture routinely collects samples as part of a monitoring program for fumonisins in maize. In 2021, 386 samples were collected and tested using a commercial lateral flow immunoassay [42]. From these samples, 64 were selected for evaluation here. These included samples with non-detectable levels as well as levels that would exceed the FDA’s regulatory guidance. Because of this, the samples tested here are not statistically representative of Illinois maize grown in 2021. Rather, they were biased to provide a sample set useful for testing the portable MS. Comparisons between sample extracts tested with the portable MS and those tested with an LC-MS are depicted in Figure 5.

The two different detection systems showed good agreement. With FB_1_ the first order regression equation was [LC-MS] = 0.117 mg/kg + 0.9124[Portable MS], r^2^ = 0.9132. For combined FB_2_/FB_3_ the equation was [LC-MS] = 0.015 mg/kg + 0.7944[Portable MS], r^2^ = 0.9481. For total FB the equation was [LC-MS] = 0.122 mg/kg + 0.8762[Portable MS], r^2^ = 0.9294.

**Figure 6:**
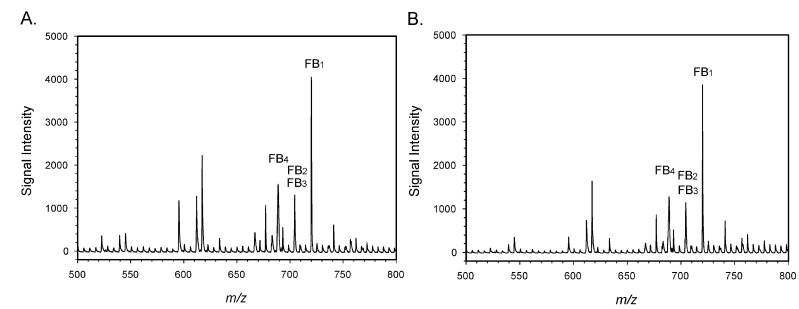
Samples of naturally contaminated maize. (**A**) a certified reference material containing 2.2 mg/kg FB_1_, 0.6 mg/kg FB_2_, and 0.3 mg/kg FB_3_ (total: 3.1 mg/kg); (**B**) sample C126 containing 2.2 mg/kg FB_1_, 1.2 mg/kg FB_2_/FB_3_. FB_4_ was added as an internal standard.

The portable MS was also used to determine the levels of FBs in two maize reference materials. The assigned (reference) values and the empirical values found using the portable MS are listed in Table 2. Spectra associated with the reference material M1811-100 and a sample of Illinois maize with similar toxin levels (C126) are depicted in Figure 6.

## 3. Discussion

Unlike immunoassays, which indirectly detect mycotoxins based on their interaction with an antibody, MS-based methods offer the potential of direct detection of a physical property of the toxin itself. While the selectivity of immunoassays is derived from the antibodies used, with MS instruments, the selectivity results in large measure from the mass analyzer. MS instruments are often viewed as enabling greater confidence in the analytical result. This is certainly true for high-resolution mass spectrometers or those instruments where tandem MS can be performed. Compact single and dual quadrupole mass spectrometers have found widespread use within and outside analytical laboratories. However, the volumes of gas needed, along with the vacuums needed for the mass analyzers, have posed challenges for making truly portable, scanning, mass spectrometers. In this report, two compact mass spectrometers were used, one of which was laboratory-based (the Waters QDa) and one of which was transportable (the BaySpec Portability). Here, we have made the distinction between ‘compact’ and ‘portable’ based on the different needs of the two instruments for power and inlet gas. The QDa is a compact unit that uses a full size external vacuum pump and requires a source of compressed nitrogen. The Portability uses much smaller, internal, vacuum pumps and uses air as the inlet gas.

While portable MS instruments, in particular GC-MS instruments, have found widespread use in many fields, applications to mycotoxins have not been reported. One reason may be the low volatility of most mycotoxins. For the trichothecene mycotoxins, such as deoxynivalenol (DON), GC-MS methods often require derivatization to improve volatility. The need to derivatize reduces one of the advantages of a portable instrument: the potential to be used in screening assays. Renaud et al. published a review article, wherein proof-of-concept data was presented for detecting DON as [DON + H]^+^ in a solid wheat sample using a portable MS instrument [43]. Unfortunately, we have been unable to find a follow-up to that preliminary report.

As with DON, the fumonisins are poorly volatile. Although readily amenable to ionization with electrospray sources, the fumonisins have proven to be poor candidates for ambient ionization techniques such as Direct Analysis in Real Time mass spectrometry (DART-MS) [44,45]. This is the first report describing the application of a truly portable mass spectrometer to the detection of fumonisins in maize. Our initial experiments with the instrument involved tests to determine whether an APCI source would allow detection of fumonisin standards, which it did not. Follow-up experiments included testing with an ESI source, with liquid sample introduced using a metal sample ‘loop’, which was vaporized by a thermal desorption unit prior to electrospray. This method of sample introduction, although convenient, did not yield consistent ionization. The third set of trials involved using the syringe pump of the ESI source to infuse fumonisins, which passed under the thermal desorption unit. This method of sample introduction allowed for consistent signal development.

Ideally, a field portable method would not require an extraction step or would incorporate a rapid extraction and little or no cleanup of the extract prior to determination. The preliminary report on DON in wheat referenced earlier suggested that this might be possible. Renaud et al. [43] reportedly introduced solid wheat into the instrument and detected [DON + H]^+^ at *m/z* 297. Because we were using the same instrument (the BaySpec Portability MS) we tried a similar experiment with fumonisin contaminated corn, without success. As an alternative to introducing solids into the instrument, we conducted a series of preliminary experiments using un-purified extracts of maize. The extractions used mixtures of methanol/water or acetonitrile/water that have been reported previously to extract fumonisins from maize. However, with such extracts, the matrix caused severe ion suppression, to the extent that only highly concentrated solutions could be detected. In follow-up experiments, we tested a commercial cleanup column, of a design that allows rapid removal of many contaminants, but which allows the fumonisns to pass through. While such columns visibly improved the purity of the extract, excess matrix remained that continued to cause signal suppression. Next, immunoaffinity columns, which retained the fumonisins, rather than the impurities, were investigated.

While the IAC did not completely prevent ion suppression (Figure 3), interferences were sufficiently removed to allow the detection of fumonisins at relevant levels. In the United States, the guidelines provided by the USFDA for fumonisins range from 2 mg/kg in degermed dry-milled corn products intended for human consumption to 100 mg/kg in the feed of poultry being used for slaughter, at 50% of the diet [9]. These guidelines refer to the sum of FB_1_, FB_2_, and FB_3_. Therefore, meeting the guidelines does not require differentiation between FB_1_, FB_2_, and FB_3_. Nevertheless, the ability to discriminate between the congeners, which generally co-occur, provides an additional level of confidence not provided by immunoassays. To determine whether the portable MS was sufficiently sensitive to detect fumonisins at the lower levels associated with human foods, recovery studies were conducted. Results (Table 1, Figure 2) suggested the method was sufficiently sensitive to detect fumonisins in maize at levels relevant to the regulatory guidance. Average recoveries were acceptable, ranging from 93.6% to 108.6%. However, variability was relatively high, with RSDs ranged from 12.0 to 29.8%. The USDA Federal Grain Inspection Service has published test performance specifications for quantitative fumonisin test kits, applicable to kits such as the AgriStrip Fumonisin WATEX [46]. The criteria specify maximum RSDs ranging from 13% to 18%. By this metric the portable MS did not meet the standard at the highest and lowest spiking levels. Clearly then, reproducibility is the area where the portable MS method requires the most improvement.

To determine whether the technique could meet the EU criteria for a screening assay, spiked and unspiked samples were analyzed, in quadruplicate, over 6 days. The spiking level selected was 0.57 mg/kg of total fumonisins (0.38 mg/kg FB_1_ and 0.19 mg/kg combined FB_2_ and FB_3_). This screening target concentration (STC) was used to estimate cut-off values (Figure 4). The cut-off values, which signify the level at which there is a 5% chance of a false negative result, were equivalent to 0.22 mg/kg FB_1_, 0.13 mg/kg of combined FB_2_ and FB_3_, and 0.42 mg/kg of total fumonisins. These results suggest that the portable MS method was capable of being used to screen for fumonisins at levels at, or below, the regulatory guidelines.

The portable MS was also applied to the analysis of 64 samples of maize collected from sites throughout the state of Illinois, and to two maize reference materials [42]. For comparison, the naturally contaminated samples were also tested by LC-MS with a compact dual quadrupole instrument (Waters QDa). Good comparisons were found between the two detection techniques, for FB_1_ alone, FB_2_ and FB_3_ in combination, and total fumonisins (Figure 5A–C, respectively). The slopes of the regression lines, 0.9124, 0.7944, and 0.8762, suggest that the portable MS was yielding results slightly higher than the LC-MS for FB_1_, and about 26% higher for FB_2_. The result was an overall overestimation of about 14% for total FBs with the portable MS compared to the LC-MS.

The Illinois samples were screened initially using lateral flow test strips. The method using the strips was rapid; approximately 10 min when starting with ground maize. The portable MS method was not as rapid; circa 70–80 min for individual samples. The longer analysis time resulted in large part from the longer sample pre-treatment for the MS method. This included a longer extraction step (30 min vs. 2 min), and cleanup with the immunoaffinity column (35–45 min vs. a 2 min ‘settling’ step for the test strips). The final, determinative, steps were similar (5 min vs. 3 min).

Two maize reference materials, one at a modest fumonisin concentration (3.1 mg/kg) and one at a high fumonisin concentration (37.1 mg/kg) were also examined. For the material containing less fumonisin, the two methods compared quite well (Table 2). The level of FB_1_ found was slightly lower (2.0 vs. 2.2) and the combined FB_2_/FB_3_ level was slightly higher (1.1 vs. 0.9) compared to the reference values, while the total fumonisin content was the same (3.1). Similarly, for the material containing more fumonisin, the portable MS tended to slightly underestimate FB_1_ and overestimate FB_2_/FB_3_. However, for total fumonisins the portable MS reported a value of 87% of the assigned (reference) value. The reason for the greater difference at higher fumonisin concentrations is unclear, but may be related to the capacity of the immunoaffinity columns and the relative binding of FB_1_, FB_2_, and FB_3_ to them.

## 4. Conclusions

Rapid, accurate, decisions about whether or not a commodity is contaminated with fumonisin need to be made at locations outside of traditional analytical laboratories. Currently, much of this testing is done indirectly, by immunoassay. MS instruments enable direct detection, as well as the measurement of multiple congeners of the fumonisins, increasing confidence in the analytical result. The increased availability of portable mass spectrometers enables the application of MS in locations where previously it would have been impossible. This study demonstrated the ability of a portable MS instrument to be used to measure, fumonisins in maize at concentrations relevant to USFDA guidance levels.

## 5. Materials and Methods

### 5.1. Materials

Except where noted otherwise, deionized water (Nanopure II, Thermo Scientific, Waltham, MA, USA) was used in the preparation of all reagents. Solid FB_1_ was provided by Robert Eppley (USFDA). Solid FB_2_, FB_3_, and FB_4_ used in spiking studies were prepared at the National Center for Agricultural Utilization Research (Peoria, IL, USA) by Steve Poling and Ronald Plattner [47]. Analytical standard mixture of FB_1_, FB_2_, and FB_3,_ product number TAS-MM19LZ1-2, lot number 210128R-22138 was purchased from Trilogy Analytical Laboratory (Washington, MO, USA). Also purchased from Trilogy were two maize certified reference materials (CRMs) containing fumonisins, products TR-MT100 (lot MTC-9999D) and TCRM-M1811-100 (lot 121174(3.1M)). Phosphate buffered saline (PBS) was prepared as a mixture of 0.14 M NaCl, 8.5 mM Na_2_HPO_4_, 1.5 mM KH_2_PO_4_, and 2.7 mM KCl in H_2_O. FumoniTest WB immunoaffinity columns (IAC), product number G1060 were purchased from VICAM (Waters Corporation, Milford, MA, USA). All other chemicals were reagent grade or better and purchased from major suppliers.

### 5.2. Samples

Maize samples were collected by the Illinois Department of Agriculture (ILDA), division of Agricultural Products Inspection (Springfield, IL, USA). Samples (386) were collected in autumn of 2021 from a variety of sites throughout the state of Illinois. The samples were ground, and then tested for total fumonisins using a commercial rapid test kit (AgraStrip Fumonisin WATEX, Romer Labs, Inc., Newark, DE, USA). After testing, samples were accumulated and approximately 100 g subsamples of ground maize were shipped to the USDA-NCAUR (Peoria, IL, USA). Samples were received in December 2021 and were stored at −20 °C until 2–3 days before extraction. The results from the rapid test were used to select a subset of 64 samples to test using the portable mass spectrometer and LC-MS. Samples were selected in order to provide a range of fumonisins from non-detectable with the rapid test (<0.25 mg/kg) to levels exceeding USFDA guidelines. No samples of extremely high fumonisins were found and therefore, with the exception of one of the CRMs, were not included in the method validation.

### 5.3. Extraction and Cleanup

Sodium chloride (1.5 g) was added to 15 g of ground maize. Next, 30 mL of methanol/water (MeOH/H_2_O, 4 + 1 *v/v*) was added and the mixture was shaken for 30 min on a wrist-action shaker (Burrell Corporation, Pittsburg, PA, USA). The mixture was filtered through a Whatman 2 V filter (Cytiva, Buckinghamshire, UK) until 10 mL was obtained. The extract was diluted with 40 mL of PBS, causing precipitation. The diluted extract was filtered once more, through a Whatman 934-H glass fiber filter(Cytiva). Then, 10 mL of the filtered extract was applied to a VICAM FumoniTest WB column, at a flow rate of 1–2 drops/s. The column was rinsed with 10 mL of deionized water, also at 1–2 drops/s. The fumonisins were eluted with 1 mL of MeOH, followed by 1 mL of 0.2% (*v/v*) aqueous formic acid. The purified extracts contained the equivalent of 0.5 g maize/mL extract. As an internal standard, the purified extracts were spiked with FB_4_ solution to a concentration of 1.5 µg/mL (52 µL of 60 µg/mL stock in acetonitrile/H_2_O).

For determination of matrix effects, calibrants were prepared either in acetonitrile/H_2_O/formic acid solution (84 + 16 + 0.1, *v/v/v*) or in purified extracts of low-fumonisin maize. Initially the calibrants were based upon fumonisin standards prepared at NCAUR. Later experiments showed these calibrants did not match those obtained from Trilogy. To align the two sets of calibrants, the NCAUR standards were recalibrated relative to the Trilogy standard. This was essential for consistency and because of the large amounts of FB_1_, FB_2_, and FB_3_ needed for the spiking and recovery studies, for which we could not afford to use the Trilogy standard. Calibrants were prepared over the range of 0.061 to 3.052 µg/mL FB_1_, 0.011 to 0.582 µg/mL FB_2_, and 0.018 to 0.895 µg/mL FB_3_. The total (FB_1_ + FB_2_ + FB_3_) ranged from 0.091 to 4.528 µg/mL. Maize was spiked over the range of 0.38 to 3.81 µg/g FB_1_, 0.19 to 1.85 FB_2_ and FB_3_ (combined). The total FB was spiked over the range of 0.57 to 5.66 µg/g. Spiking was accomplished by adding standard fumonisins, in acetonitrile/H_2_O to ground maize, with the solvent allowed to evaporate overnight before extraction. To assist in determination of cut-off values, samples of unspiked maize (*n* = 24) and maize spiked with a total of 0.57 µg/g fumonisins (*n* = 24) were analyzed on 6 separate days. This was done to meet the criteria of the European Commission, which specifies a minimum of 20 samples analyzed on 5 separate days [41].

### 5.4. Instrumentation

The portable mass spectrometer was a model Portability manufactured by BaySpec (San Jose, CA, USA). The mass analyzer is a linear ion trap, equipped with an inlet for atmospheric sampling. The source was a thermal desorption electrospray ionization (TD-ESI) unit, equipped with two routes for sample introduction: a metal inoculation loop (circa 2 µL) and a syringe pump through which liquids could be infused. The unit does not require the use of external gasses (e.g., nitrogen or helium). For these experiments, purified extracts were infused into the thermal desorption unit at a rate of 2 µL/min. Source parameters were as follows: positive mode; heater: 225 °C; sample pump (air pump): 60%; high voltage: +2.8 kV. Settings for the mass analyzer were as follows: mass center: *m/z* = 721; mass tolerance: 5; spectrum average: 3; running average: 0. Spectra were collected over the *m/z* range from 50 to 850. The instrument was tuned using purified FB_1_. The tune parameters were: AC start: 3.30; AC stop: 25.0; AC frequency: 362.50; AC phase: 3.0; Rf level: 460.0; Trap bias: 2.50; Trap entry: 5.20; Trap exit: 30.10.Continuous monitoring was done at the mass center. Signals from *m/z* 690 were used to determine FB_4_ and signals from *m/z* 706 were used to determine FB_2_ and FB_3_.

For comparison, purified maize extracts were also tested with an LC-mass spectrometric (LC-MS) method. The LC was a Dionex Ultimate 3000 system with Chromeleon 7 software (Dionex Corporation, Sunnyvale, CA, USA). The column was a Phenomenex Kinetex (1.7 μm, XB-C18, 100 Å, 100 mm × 3 mm, product number 00D-4498-YO) with Security Guard ultra C18 3 mm guard cartridge, maintained at 30 °C. The mobile phase was a binary gradient of (A) acetonitrile, and (B) water/acetonitrile/formic acid (92 + 8 + 0.25 *v/v/v*) adjusted to pH 4.0. Flow rate was 0.7 mL/min, or which half was directed to the mass spectrometer and half to a photodiode array (PDA) detector. The initial mixture was 90% B. At 3 min this was reduced to 75% B, at 6 min it was reduced to 70% B, and at 10 min to 45%. At 13 min the mixture was returned to 90% B. Run times were 20 min. Injection volume was 20 μL The PDA detector was set to monitor at 202 nm. The MS was a Waters model QDa, (Waters Corporation, Milford, MA, USA) operated in positive electrospray ionization (ESI) mode, with continuous single ion monitoring of the *m/z* of 690.4 (FB_4_), 706.4 (FB_2_ and FB_3_), and 722.7 (FB_1_). Operation was in positive mode electrospray injection. Parameters were as follows: detector gain: 1; temperature probe: 425 °C; capillary voltage: 1.5 kV; and data collection rate: 15 Hz. Retention times were 5.8, 7.9, 9.4, and 11.4 min for FB_1_, FB_3_, FB_2_, and FB_4_, respectively.

### 5.5. Data Analysis, Limits of Detection, Quantitation, and Cut-Off Value

Internal standards are an established mechanism for improving the reproducibility of mass spectrometric methods, with isotopically labeled standards commonly used. While extremely useful, when added to every sample they can be very expensive. Instead, we chose to use FB_4_ as the internal standard. FB_4_ was added to all of the purified extracts (standards and samples) to a level of 1.5 µg/mL, equivalent to 3 µg/g in maize. Signals from the other fumonisins were divided by the FB_4_ signal to obtain relative response ratios. For analysis of samples with the portable MS, matrix-matched calibration curves were prepared and data were fit using a linear function. For analysis of samples by LC-MS, matrix-matched calibration curves were prepared and data were fit using a logistic dose–response function (Equation 8013, TableCurve, SYSTAT Software, Inc., Richmond, CA, USA).

For the portable MS, limits of detection (LOD) were calculated by measuring the response ratios of each of the fumonisins obtained from 20 unspiked samples, tested as part of the calibration curves on 20 separate days. The LOD was determined as the concentration calculated to give a response 3 standard deviations above that of the average of the unspiked samples. The limit of quantitation (LOQ) was determined as the concentration calculated to give a response 10 standard deviations above that of the average of the unspiked samples.

The European Union (EU) has established performance criteria of mycotoxin testing, as described in Commission Regulation EU No.519/2014 [41]. For screening methods with a response proportional with the mycotoxin concentration the cut-off level is calculated based upon the equation:

Cut-Off value = R_STC_ − t-value_0.05_ × SD_STC_ (1)

where STC is the screening target concentration, the concentration at which the assay will be used to screen samples. In this case, the STC was the lowest spiking concentration (0.57 µg/g total fumonisins). The R_STC_ was the average response from 24 samples containing fumonisins at the STC. The t-value was the one-tailed t-statistic for a rate of false negative results of 5% (1.714 for 24 samples). The SD_STC_ was the standard deviation in the signal ratios for the 24 spiked samples.

## Figures and Tables

**Table 1:** Recovery of fumonisins from spiked maize.

Toxin	Spiking Level (mg/kg)	Recovery (% ± SD) ^1^	RSD (%)	n
FB_1_	0.38	101.6 ± 19.2	18.9	24
	0.76	101.7 ± 15.7	15.4	7
	1.91	93.6 ± 11.6	12.4	7
	3.81	96.2 ± 28.7	29.8	7
FB_2_ & FB_3_	0.19	108.6 ± 29.0	26.7	24
	0.37	107.0 ± 17.5	16.4	7
	0.92	99.2 ± 11.9	12.0	7
	1.85	105.8 ± 23.4	22.1	7
Total FB	0.57	106.7 ± 18.8	17.6	24
	1.13	104.4 ± 14.8	14.2	7
	2.83	95.8 ± 11.6	12.1	7
	5.66	99.5 ± 27.0	27.1	7

^1^ SD: standard deviation, RSD: relative SD, n: number of replicate samples.

**Table 2:** Analysis of two reference materials with the portable MS.

Reference Material	Toxin	Assigned Value (mg/kg)	Reported SD ^1^	Value Found with the Portable MS (mg/kg)	SD (*n* = 3)
M1811-100	FB_1_	2.2	0.2	2.0	0.1
	FB_2_	0.6	0.1	1.1	0.04
	FB_3_	0.3	0.1		
	Total FB	3.1	NR	3.1	0.12
MT-100	FB_1_	28.3	NR	21.1	3.4
	FB_2_	7.1	NR	11.3	1.0
	FB_3_	1.7	NR		
	Total FB	37.1	4.2	32.3	4.1

^1^ SD: standard deviation, NR: not reported.

## Data Availability

Not applicable.

## Supplementary Materials

The following supporting information can be downloaded at: https://www.mdpi.com/article/10.3390/toxins14080523/s1, Figure S1: Matrix-matched calibration lines for fumonisins using the portable MS. (A) FB_2_ and FB_3_, combined; (B) total fumonisins (FB). Lines represent first order regression. For FB_2_ & FB_3_, Y = 0.1203 + 0.8478 (X), r^2^ = 0.9997. Total FB fit the equation Y = 0.1477 + 0.8598(X), r^2^ = 0.9992.
