# Supplementary material for: Measurement of Fumonisins in Maize Using a Portable Mass Spectrometer
Source: Toxins (Basel). 2022 Jul 30;14(8):523. doi: 10.3390/toxins14080523 (PMC9412256; doi:10.3390/toxins14080523)
Supplement: Supplementary file 1 [file toxins-14-00523-s001.zip › toxins-1828954-supplementary.pdf]

toxins

Supplementary Materials: Measurement of Fumonisins in Maize Using a Portable Mass Spectrometer

Chris M. Maragos, Kristin Barnett, Luke Morgan, Martha M. Vaughan and Kristal K. Sieve

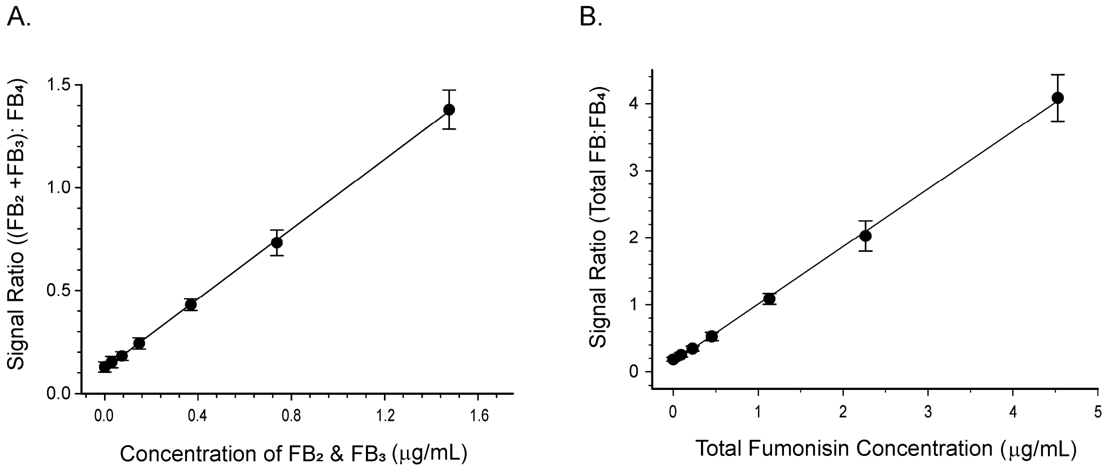

Figure S1. Matrix-matched calibration lines for fumonisins using the portable MS. (**A**) FB_2 and FB_3, combined; (**B**) total fumonisins (FB). Lines represent first order regression. For FB_2 & FB_3, Y = 0.1203 + 0.8478 (X), $r^2 = 0.9997$. Total FB fit the equation Y = 0.1477 + 0.8598(X), $r^2 = 0.9992$.
